# Joint Associations of Residential Ambient PM_2.5_ Components, Nutritional Indices, and Leisure-Time Physical Activity with Lower Estimated Glomerular Filtration Rate Among Subway Workers

**DOI:** 10.3390/nu18152580

**Published:** 2026-08-06

**Authors:** Xiaomeng Tan, Wanyan Zhang, Yingri Zhang, Lv Shang, Fang Ye, Jian Hou, Li Liu, Zijian Zhao, Zhenyu He

**Affiliations:** 1 School of Management, Zhengzhou University, Zhengzhou 450001, China; tanxiaomeng@gs.zzu.edu.cn; 2 School of Kinesiology and Physical Education, Zhengzhou University, Zhengzhou 450001, China; 3 Department of Epidemiology and Biostatistics, College of Public Health, Zhengzhou University, Zhengzhou 450001, China; wanyan0611@stu.zzu.edu.cn (W.Z.); yrzhang0@163.com (Y.Z.); 13667176505@163.com (J.H.); 4 Wuhan Center for Disease Control and Prevention, Wuhan 430024, China; 18627097901@163.com; 5 School of Public Health, Tongji Medical College, Huazhong University of Science and Technology, Wuhan 430074, China; yefang@hust.edu.cn (F.Y.); liul2012@hust.edu.cn (L.L.)

**Keywords:** fine particulate matter, kidney function, Geriatric Nutritional Risk Index, Prognostic Nutritional Index, subway workers

## Abstract

**Background**: Long-term exposure to fine particulate matter (PM_2.5_) and its components, as well as lower nutritional scores or irregular leisure-time physical activity, has been individually linked to reduced kidney function. However, the joint associations of PM_2.5_ component mixture combined with nutritional scores or leisure-time physical activity with estimated glomerular filtration rate (eGFR) remain underexplored, especially among subway workers. **Method**: This study included 8477 subway workers in Wuhan, China. PM_2.5_, and component data were obtained from the Tracking Air Pollution in China (TAP) dataset. Leisure-time physical activity was assessed using a self-administered questionnaire adapted from the International Physical Activity Questionnaire (IPAQ). Kidney function was evaluated by creatinine-based eGFR. Nutritional indices of Geriatric Nutritional Risk Index (GNRI) and the Prognostic Nutritional Index (PNI) were calculated. Generalized linear models were used to examine the associations of PM_2.5_ and each of its components or nutritional indices with lower creatinine-based eGFR. The associations of PM_2.5_ component mixture with creatinine-based eGFR were evaluated using WQS, QGC, and BKMR models. Joint associations of PM_2.5_ component mixture with nutritional indices or leisure-time physical activity were further explored. **Results**: Negative associations of individual PM_2.5_ components and their mixture with eGFR values were observed. WQS and QGC models showed that the mixture of PM_2.5_ components was associated with an increased risk of lower creatinine-based eGFR, and their corresponding ORs (95%CI) were 1.183 (1.106, 1.266) and 1.183 (1.083, 1.291), respectively. BKMR analysis further showed the overall effect on eGFR values in relation to a 5-percentile increase in the PM_2.5_ component mixture was −0.045 (−0.068, −0.022), compared with their levels fixed at their respective medians. Meanwhile, the probability of lower creatinine-based eGFR increased with rising quantiles of PM_2.5_ component mixture concentration. Notably, differential associations were observed across subgroups classified by nutritional indices or leisure-time physical activity, but no significant interaction was observed. **Conclusions**: Long-term exposure to residential ambient PM_2.5_ components or their mixture is associated with lower creatinine-based eGFR. This different association was observed among subgroups classified by nutritional indices or leisure-time physical activity, which may inform hypothesis generation for future studies.

## 1. Introduction

Chronic kidney disease (CKD) has posed a global public health concern. Results from the global burden of disease study 2019 indicate that the CKD burden remains high across various income region, particularly in low- and middle-income countries (LMICs) [1]. In China, prevalence and mortality rates of CKD increased from 6.7% to 10.6%, and from 8.3/100,000 to 13.8/100,000, respectively, between 1990 and 2019 [2]. LIMCs may bear a greater burden of CKD than high-income countries, leading to higher treatment costs and poorer quality of life, which can be attributed to disparities in burden of communicable and non-communicable diseases, levels of air pollution, and other socioeconomic factors [1,3,4]. Long-term exposure to air pollution, particularly to fine particulate matter (PM_2.5_), has been identified as an emerging risk factor for CKD [5,6]. In recent years, accumulating evidence has indicated that long-term exposure to PM_2.5_ is associated with renal function decline and an increased risk of CKD [7,8,9,10,11]. Previous studies have linked PM_2.5_ exposure to impaired renal function across different populations. For instance, a Chinese national birth cohort of 2,546,047 young adults reported a significant association between PM_2.5_ and reduced renal function [12]. Similarly, an 18-year longitudinal study among Asian children and adolescents similarly demonstrated that long-term PM_2.5_ exposure was associated with a lower estimated glomerular filtration rate (eGFR) and a higher incidence of CKD [9]. Additionally, Adgate et al. found that airborne particulate matter exposure was associated with CKD risk in male sugarcane workers aged 18–57 in Guatemala [13]. However, limited studies have assessed the short-term effect of PM_2.5_ and its components on renal health among elderly individuals and healthy adults [14,15,16], and research focusing on adverse renal effects of long-term exposure to PM_2.5_ and its components remains scarce. Furthermore, identifying the major contributors to those effects is also an urgent research priority.

Malnutrition may arise from an imbalance between nutritional requirements and intake, leading to metabolic alterations, functional impairment, and loss of body mass [17]. The prevalence rate of malnutrition varies considerably across populations, such as community-dwelling older adults and hospitalized elderly individuals [17,18]. Numerous studies have suggested that malnutrition is related to renal dysfunction in community-dwelling older adults and is further associated with the progression of CKD among patients with heart failure [19,20,21]. For instance, one cross-sectional study assessing the association of nutritional indices with kidney function among community-dwelling elderly individuals aged 75 years and older showed that low serum albumin levels and nutritional status were positively associated with eGFR decline [22]. Another study conducted in older adults without advanced kidney disease found that malnutrition was related to an increased risk of kidney function decline and CKD [19]. In addition to nutritional factors, several studies have reported interactions between air pollution and physical activity. The benefits of physical activity may be diminished under high levels of air pollution, potentially attributed to increased exposure to PM_2.5_ [23,24,25]. However, other studies have found no such interaction [26,27], or have even suggested that physical activity may reduce the adverse effects of air pollution [25,28,29]. However, the joint associations of nutritional factors, physical activity and air pollution with kidney function in the young working-age population remain largely unclear.

In recent years, nutritional indices such as the Geriatric Nutritional Risk Index (GNRI) and Prognostic Nutritional Index (PNI) have been increasingly used in clinical and epidemiological settings as practical and easily calculable screening tools [30,31]. Both indices are derived from routinely available laboratory and anthropometric parameters, including serum albumin, body weight, and lymphocyte count, making them particularly suitable for large-scale population-based studies in which comprehensive dietary assessments are often infeasible. Although previous studies have explored individual association of air pollution, nutritional indices with kidney function, their joint associations remain poorly understood. Notably, recent work has extended the application of these indices beyond elderly or hospitalized patients to younger populations [32]. Accordingly, in this exploratory cross-sectional study conducted among subway workers in Wuhan, we employed GNRI and PNI as descriptive stratifying variables based on routinely collected health examination data, rather than as validated comprehensive measures of nutritional status. Specifically, we aim to: (1) assess the associations of nutritional indices (GNRI or PNI), as well as PM_2.5_ and its components derived from the Tracking Air Pollution in China (TAP) Data with lower creatinine-based eGFR; (2) examine the interactive effects of lower nutritional indices, leisure-time physical activity, and air pollution on lower creatinine-based eGFR. Given the exploratory nature of this cross-sectional design, our findings underscore the urgent need for longitudinal studies with validated nutritional assessments to determine whether nutritional indices may serve as potential biomarkers for lower creatinine-based eGFR in the context of air pollution exposure among young working-age populations.

## 2. Material and Methods

### 2.1. Study Population

From December 2018 to May 2019, a total of 11,960 subway workers were enrolled from Wuhan Metro Group Co., Ltd. (Wuhan, China). A questionnaire was used to collect information regarding individual characteristics (age, gender, body mass index (BMI)), socioeconomic status (e.g., education level and marital status), lifestyle (smoking status, drinking status and leisure-time physical activity, etc.), as well as personal history of chronic diseases. After excluding individuals with missing information on individual characteristics, lifestyle, routine blood and biochemical indices, as well as air pollution exposure levels, a total of 8477 individuals were included in the final analysis. The participant flow diagram is presented in Figure S1.

The socioeconomic status (education levels, marital status), lifestyle (smoking and drinking status, as well as leisure-time physical activity) and personal history diseases of hypertension, dyslipidemia and type 2 diabetes were described in our previous study [33]. CVD risk factors were defined as including one of the following: hypertension, dyslipidemia, or type 2 diabetes. Briefly, leisure-time physical activity was assessed using a self-administered questionnaire adapted from the IPAQ short form, which was supplemented with culturally specific items for the Chinese population (e.g., walking, biking, jogging, dancing, and ball sports). Frequency (days per week) and average duration (minutes per session) were recorded for each reported activity type. Metabolic equivalent (MET) values were assigned according to the Compendium of Physical Activities [34,35]. Total leisure-time physical activity was calculated as MET coefficient × duration × frequency and expressed as MET-minutes/week. Leisure-time physical activity was defined as accumulating at least 150 min/week of moderate-intensity activity, 75 min/week of vigorous-intensity activity, or an equivalent combination of both [36]. This study was approved by the Wuhan Center for Disease Control and Prevention Ethics Committee (ethics approval No. 2018042). All individuals provided written informed consent prior to their participation in the study.

### 2.2. Estimation of PM_2.5_ and Its Component Concentrations

Concentrations of PM_2.5_ and its five components (sulfate (SO_4_^2−^), nitrate (NO_3_^−^), ammonium (NH_4_^+^), organic matter (OM), and black carbon (BC)) were derived from the TAP in China database (http://tapdata.org.cn/). The estimation methods have been described elsewhere [37,38]. Briefly, the daily PM_2.5_ concentrations were estimated at a 0.1° × 0.1° spatial resolution using a two-stage machine learning model. The model was developed using multisource data fusion, including ground-based observations, satellite-retrieved fractional aerosol optical depth (AOD), chemical transport models (CTM) simulations, and ancillary data, such as meteorological, land use, population, and elevation variables. Daily concentrations of PM_2.5_ and its five components were estimated through a multi-stage procedure. The Weather Research and Forecasting–Community Multiscale Air Quality (WRF–CMAQ) model was used to generate component-specific conversion factors (CFs). Then the extreme gradient boosting algorithm was used to correct the relative contribution of PM_2.5_ component concentrations based on the CFs. The predicted PM_2.5_ components showed good agreement with the surface measurements, with R^2^ values ranging from 0.67 to 0.80. Each participant’s residential address was geocoded for latitude and longitude using Google Maps (*arcgis* 10.8.1), and the air pollutant concentrations were extracted from the nearest grid cell containing that address. In accordance with a previous study [39], the 3-year average concentrations of PM_2.5_ and its components were used to represent long-term exposure.

### 2.3. Assessment of Nutritional Indices

All individuals were required to fast for at least 8 h prior to their health examination at the center designated by Wuhan Metro Group Co., Ltd. Venous blood samples were collected from each individual into ethylenediaminetetraacetic acid (EDTA) anticoagulation tubes by trained nurses, and routine blood and biochemical measurements were performed on the same day using an Automatic Biochemical Analyzer. Serum creatinine concentration was measured using the sarcosine oxidase assay on an automatic biochemical analyzer. Calibration of this method is traceable to NIST Standard Reference Material 914a, which serves as a primary reference standard. The GNRI was calculated using the following equation: GNRI = 1.489 × serum albumin (g/dL) + 41.7 × (actual body weight/ideal body weight), where the actual-to-ideal body weight ratio was capped at 1 for participants with overweight or obesity [30]. The PNI was calculated as follows: PNI = serum albumin (g/L) + 5 × total lymphocyte count (10^9^/L) [31].

### 2.4. Assessment of Kidney Function

Kidney function was evaluated using the creatinine-based eGFR. The eGFR values of individuals were calculated using the Chinese Modified Simplified Modification of Diet in Renal Disease (MDRD) equation as follows: eGFR = 186 × Scr^−1.154^ × age^−0.203^ × 0.742 (if participants is female) × 1.233 [40]. The eGFR analytical threshold of 90 mL/min/1.73 m^2^ was selected based on the Kidney Disease: Improving Global Outcomes (KDIGO guidelines), primarily to ensure sufficient subgroup sample size for stratified analyses in this relatively healthy occupational cohort. However, single creatinine-based measures lack confirmatory validation, which limits their utility for individual clinical inference. Moreover, this threshold alone does not establish a clinical diagnosis of CKD, as that requires either persistent eGFR reduction or ancillary evidence of kidney damage.

### 2.5. Statistical Analysis

Categorical variables were expressed as numbers (percentages), and their distributions were compared using the Chi-square test across gender. Normally and non-normally distributed continuous variables were presented as mean (standard deviation, SD) and median (interquartile range, IQR), respectively. Between-gender comparisons were performed using Student’s *t*-test and the Mann–Whitney U test for normally and non-normally distributed variables, respectively.

To minimize potential confounding, the covariates were selected based on prior knowledge [41,42] and a directed acyclic graph (DAG) constructed using DAGitty (Figure S2). The DAG identified age, sex, BMI, smoking status, drinking status, annual family income, education level, marital status, and leisure-time physical activity as the minimally sufficient adjustment set (MSA) for estimating the effect of PM_2.5_ and its components on kidney function. Although CVD risk factors were not part of the MSA, we additionally adjusted for them in a separate model to account for underlying health vulnerabilities and nephrotoxic effects. Consequently, our fully adjusted model included all MSA covariates plus CVD risk factors. Given the moderately high correlations between PM_2.5_ and its components, three generalized linear models were used to evaluate the individual association of PM_2.5_, each of its components, and nutritional indices with eGFR: model 1 was unadjusted; model 2 was adjusted for age, gender; model 3 was adjusted for age, gender, socioeconomic status (education levels, annual family income, marital status), lifestyle (smoking and drinking status), leisure-time physical activity, BMI and CVD risk factors; model 4, as a sensitivity analysis, was adjusted for age, gender, socioeconomic status (education levels, annual family income, marital status), lifestyle (smoking and drinking status, leisure-time-physical activity). In addition, restricted cubic spline (RCS) regression was employed to evaluate the linearity of the dose–response relationships between creatinine-based eGFR and each exposure variable, including PM_2.5_, its components, and nutritional indices. Knots were placed at the 10, 50, and 90 percentiles of each exposure distribution, with the 10 percentile serving as the reference level.

Given that traditional statistical methods may introduce bias when modelling uncertain relationships among highly correlated multiple components in mixtures, we simultaneously applied weighted quantile sum (WQS) regression, quantile-based g-computation (QGC), and Bayesian kernel machine regression (BKMR) to comprehensively evaluate the effects of exposure to the entire chemical mixture [36,37]. In the WQS approach, a cumulative linear index was constructed by categorizing each PM_2.5_ component into quartiles, with component-specific weights representing each component’s relative contribution to the overall index. Briefly, the data were randomly partitioned into a training subset (40%) and a validation subset (60%). The training subset was used to estimate component weights and their uncertainty via a bootstrap approach, yielding a single WQS index that summarizes the overall mixture effect. The association between this index and creatinine-based eGFR was then assessed in the validation subset. This split-sample design can reduce overfitting and provide more robust estimates of the mixture effect. A key limitation of WQS is its assumption that all components exert effects in the same direction. To address this, we employed QGC, which allows mixture components to have associations in either direction. In this method, direction-specific weights (positive or negative) were assigned to each PM_2.5_ component based on its individual association with creatinine-based eGFR, and a mixture index was subsequently calculated. The association of creatinine-based eGFR in relation to each-quartile increment in the mixture index value was then assessed. For BKMR, we adopted the A-BKMR variant, which has been shown to offer high efficiency and robust performance in a previous comparative study [38]. A key advantage of the A-BKMR model is its ability to capture potential non-linear dose–response relationships and interactive effects among components. The model was fitted using a Markov Chain Monte Carlo (MCMC) algorithm with 10,000 iterations. Variable importance was evaluated via posterior inclusion probabilities (PIPs), and the overall joint effect of the mixture was assessed. Furthermore, convergence of the MCMC algorithm was diagnosed using trace plots. To further explore potential effect modification, stratified analyses were conducted to examine whether nutritional indices or physical activity modified the association between the cumulative linear index of PM_2.5_ components and creatinine-based eGFR. All data analyses were performed using the R software version 4.3.0 (R Project for Statistical Computing, Vienna, Austria). Statistical significance was set at a two-sided *p*-value < 0.05.

## 3. Results

### 3.1. Characteristics of the Study Population

**Figure 1:**
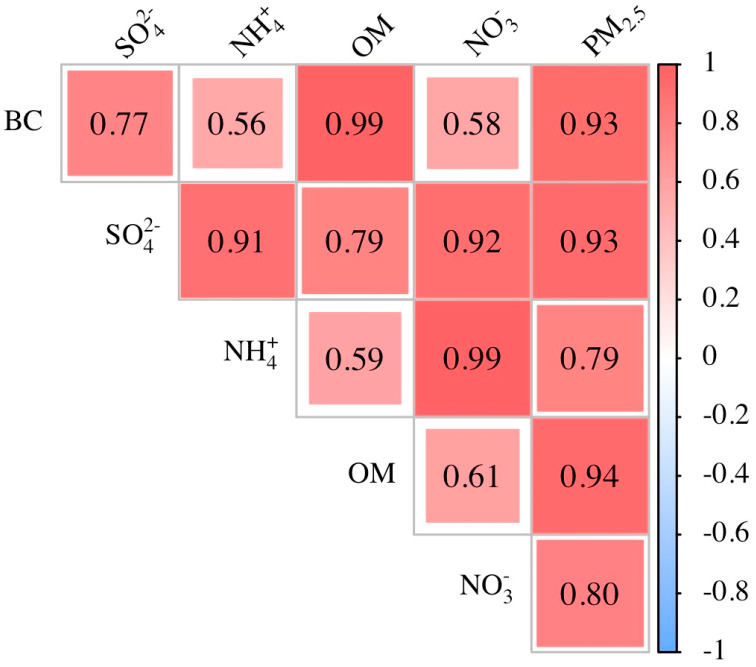
Correlation analysis of PM_2.5_ and its components between each paired air pollutant.

Table 1 shows that the mean age of individuals with eGFR < 90 mL/min/1.73 m^2^ was higher than that of those with ≥90 mL/min/1.73 m^2^. Significant differences in the distribution of selected categorical variables were observed between the two groups (eGFR < 90 vs. ≥90 mL/min/1.73 m^2^). The median levels of GNRI (108.22 vs. 109.00), and PNI (55.70 vs. 56.75) were lower among individuals with eGFR < 90 mL/min/1.73 m^2^ than among those with ≥90 mL/min/1.73 m^2^, whereas the median level of TCBI were higher among individuals with eGFR < 90 mL/min/1.73 m^2^ (all *p* < 0.05). The median concentrations of PM_2.5_, SO_4_^2−^, NO_3_^−^, NH_4_^+^, OM, and BC were 55.09, 9.72, 11.65, 7.01, 13.77, and 2.64 μg/m^3^, respectively, among individuals with eGFR < 90 mL/min/1.73 m^2^, which were higher than those among individuals with ≥90 mL/min/1.73 m^2^ (all *p* < 0.05). Figure 1 presents the correlation analysis of each paired combination of PM_2.5_ and its components, with correlation coefficients ranging from 0.59 to 0.99, indicating moderately high correlations among the air pollutants (all *p* < 0.05).

### 3.2. Associations of Air Pollutants and Nutritional Indices with eGFR

**Figure 2:**
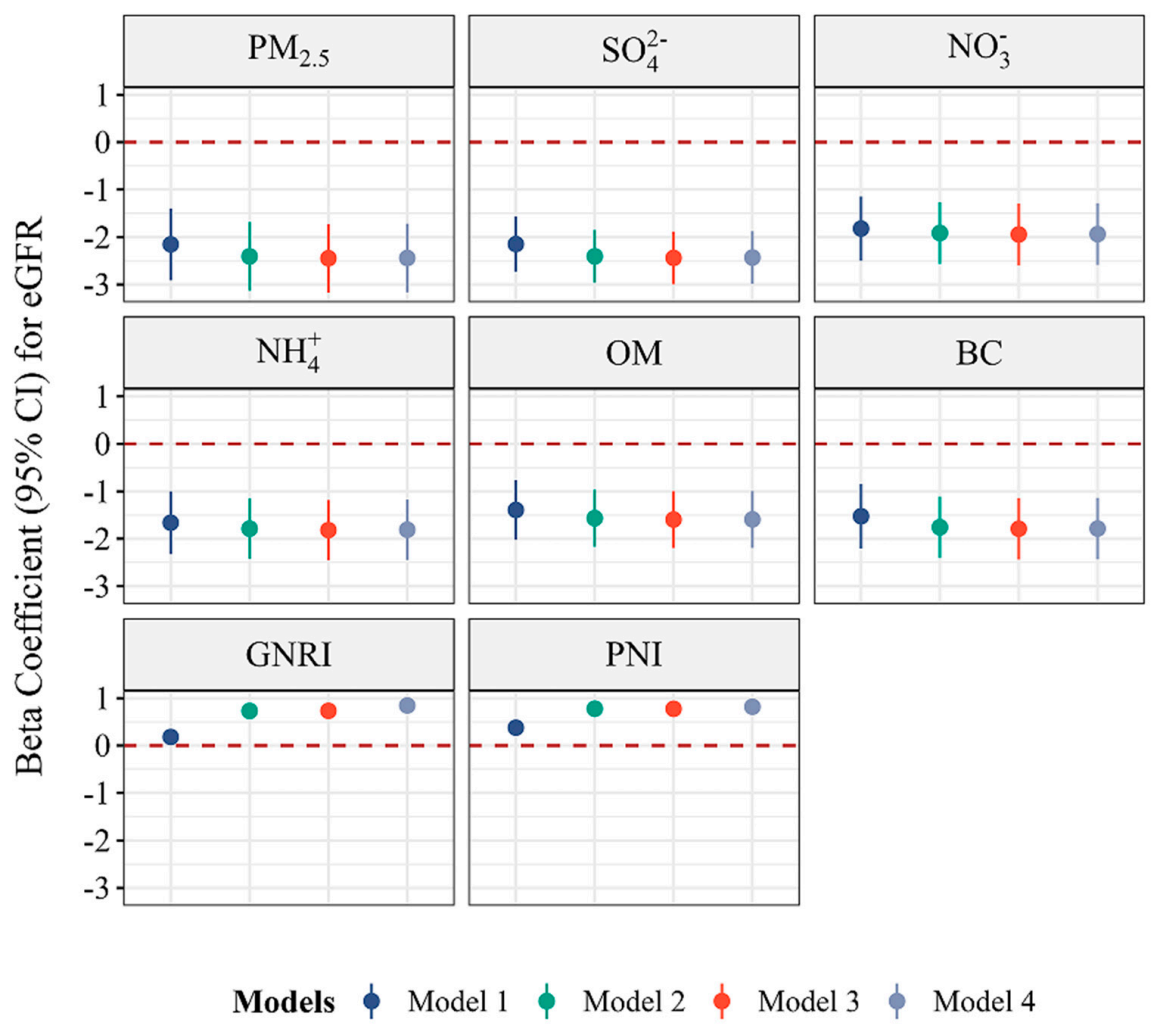
Associations of each 1-IQR increase in PM_2.5_, its components, and nutritional indices with creatinine-based eGFR indices. The estimated β coefficients and their corresponding 95% confidence intervals (95%CIs) were evaluated by the following models: model 1 was unadjusted; model 2 was adjusted for age and gender; model 3 was adjusted for age, gender, socioeconomic status (education levels, annual family income, marital status), lifestyle (smoking and drinking status), leisure-time physical activity, BMI and CVD risk factors; model 4, for sensitivity analysis, was adjusted for age, gender, socioeconomic status (education levels, annual family income, marital status), lifestyle (smoking and drinking status, leisure-time physical activity).

As shown in Figure 2, Model 1 shows that the estimated β (95%CI) values for eGFR in response to each 1-IQR increment in PM_2.5_, SO_4_^2−^, NO_3_^−^, NH_4_^+^, OM, and BC were −2.155 (−2.912, −1.397), −2.149 (−2.730, −1.568), −1.823 (−2.501, −1.145), −1.662 (−2.324, −1.00), −1.394 (−2.023, −0.765) and −1.526 (−2.204, −0.847), respectively. The estimated β (95%CI) values for eGFR in relation to each −1 unit increment in GNRI and PNI were 0.181 (0.080, 0.282) and 0.377 (0.254, 0.500), respectively. Models 2–4 show that the results did not substantially change from those of Model 1. Model 3 was used for the main analyses presented hereafter. Figure S1 shows that regarding the associations of air pollutants, nutritional indices, and physical activity with eGFR, after excluding the individuals with lower creatinine-based eGFR, the results remained largely unchanged, indicating the robustness of these findings. Table S2 shows that positive associations of PM_2.5_ and its components with serum creatinine were observed, while negative associations of nutritional indices with serum creatinine were found. Positive associations were observed between nutritional indices and blood urea nitrogen, whereas no significant associations were found between PM_2.5_ or its components and blood urea nitrogen.

**Figure 3:**
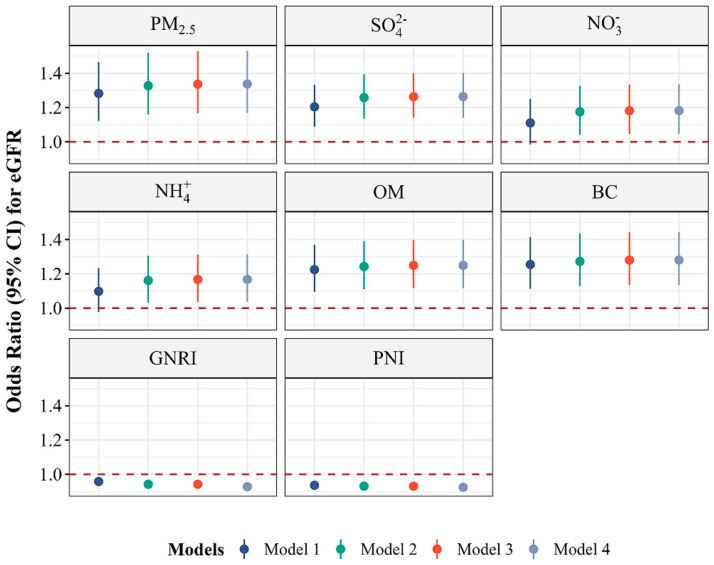
Associations of each 1-IQR increase in PM_2.5_, its components, and nutritional indices with creatinine-based eGFR indices. The estimated odd ratios (ORs) and their corresponding 95% confidence interval (95%CIs) were evaluated by the following models: Model 1 was unadjusted; model 2 was adjusted for age and gender; model 3: adjusted for age, gender, socioeconomic status (education levels, annual family income, marital status), lifestyle (smoking and drinking status), leisure-time physical activity, BMI and CVD risk factors; model 4, for sensitivity analysis, was adjusted for age, gender, socioeconomic status (education levels, annual family income, marital status), lifestyle (smoking and drinking status, leisure-time physical activity), after excluding BMI and CVD risk factors.

Figure 3 presents the associations of PM_2.5_, its components, and nutritional indices with lower creatinine-based eGFR. Model 1 presents that the estimated ORs (95%CIs) for lower creatinine-based eGFR in response to each 1-IQR increment in PM_2.5_, SO_4_^2−^, NO_3_^−^, NH_4_^+^, OM, and BC were 1.283 (1.121, 1.465), 1.204 (1.087, 1.331), 1.110 (0.983, 1.249), 1.098 (0.975, 1.230), 1.224 (1.094, 1.368), and 1.254 (1.112, 1.412), respectively. The estimated ORs (95%CIs) for lower creatinine-based eGFR in relation to each 1-unit increment in GNRI and PNI were 0.958 (0.941, 0.976) and 0.936 (0.915, 0.958), respectively. Models 2–4 display that the results were not substantially changed from the results of model 1. Model 3 was used for the main analyses presented hereafter.

**Figure 4:**
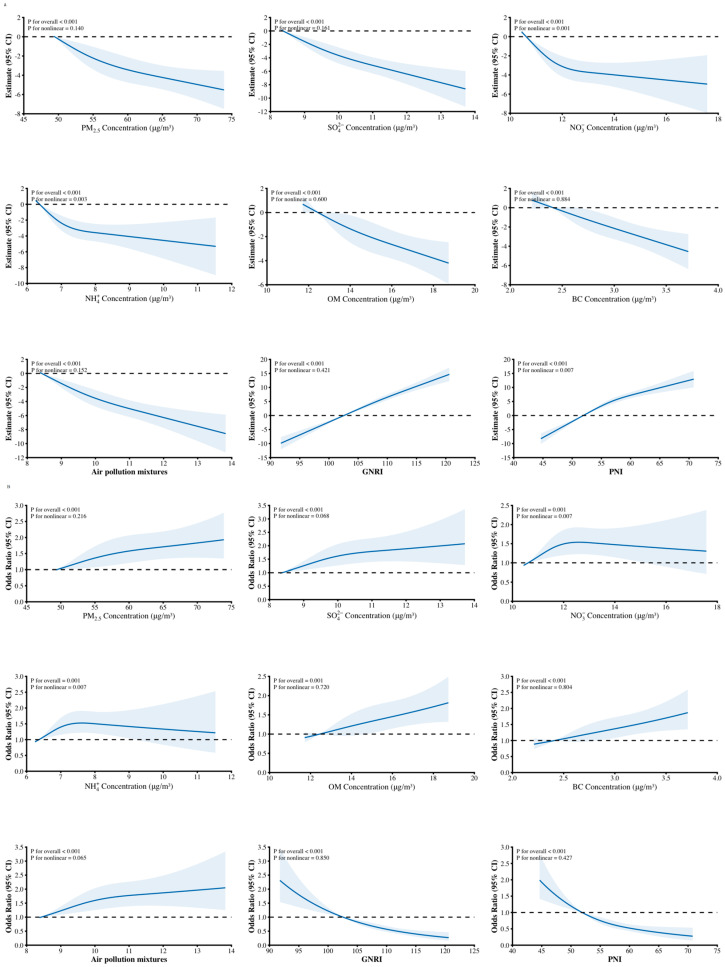
Dose–response associations of PM_2.5_, its components, and nutritional indices with eGFR (**A**) or lower creatinine-based eGFR (**B**) were analyzed by restricted cubic spline (RCS). The knots were placed at the 10th, 50th, and 90th percentiles of each exposure distribution, with the 10th percentile designated as the reference level, after adjusting for age, gender, socioeconomic status (education levels, annual family income, marital status), lifestyle (smoking and drinking status), leisure-time physical activity, BMI and CVD risk factors.

Figure 4A,B show the dose–response associations of air pollutants and nutritional indices with creatinine-based eGFR. The results indicate linear associations between air pollutants, nutritional indices and creatinine-based eGFR variations, except for NO_3_^−^, NH_4_^+^. Furthermore, a quantile regression model was used to analyze associations of NO_3_^−^ and NH_4_^+^ with eGFR, and the results show that the negative association between NO_3_^−^ or NH_4_^+^ exposure and eGFR becomes increasingly pronounced at higher exposure levels and higher eGFR quantiles (Table S3).

### 3.3. The Mixture of PM_2.5_ and Its Components with Creatinine-Based eGFR

**Figure 5:**
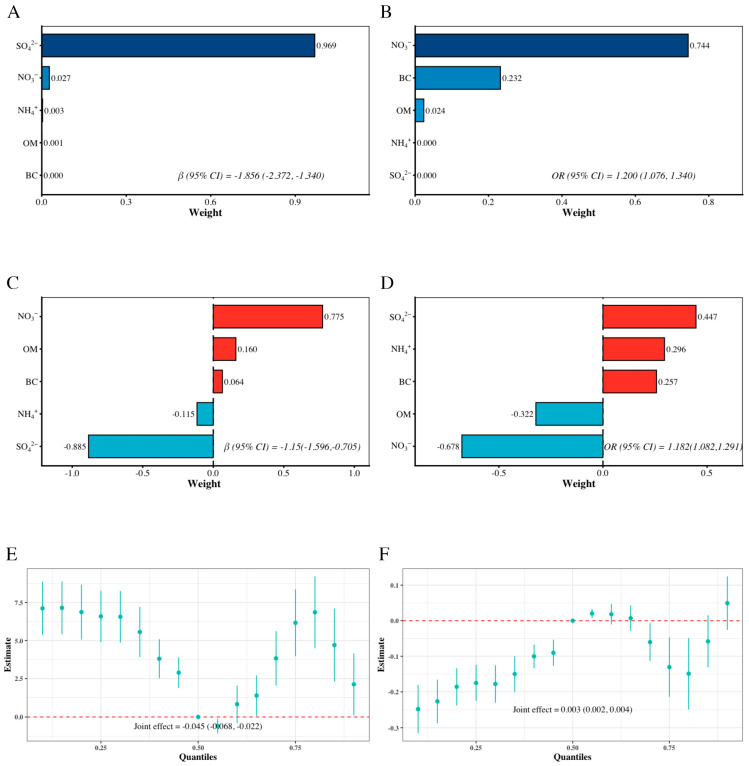
Joint associations of PM_2.5_ component mixture with eGFR and lower creatinine-based eGFR were evaluated using the WQS (**A**,**B**), QGC and BKMR models after adjusting for age, gender, socioeconomic status (education levels, annual family income, marital status), lifestyle (smoking and drinking status), leisure-time physical activity, BMI and CVD risk factors. (**A**) The WQS model showed the estimated β and the corresponding 95%CI in eGFR values in response to each-quantile increment in the mixture of PM_2.5_ components. (**B**) The WQS model showed the estimated OR and the corresponding 95%CI in lower creatinine-based eGFR in response to each-quantile increase in the mixture of PM_2.5_ components. (**C**) The QGC model showed the estimated β and the corresponding 95%CI in eGFR values in response to each-quantile increment in the mixture of PM_2.5_ components. (**D**) The QGC model showed the estimated OR and the corresponding 95%CI in lower creatinine-based eGFR in response to each-quantile increment in the mixture of PM_2.5_ components. (**E**) The BKMR model showed the estimated mean difference changes (95%CI) in eGFR values in response to each -5 percentile increment in the mixture of PM_2.5_ components, compared to all PM_2.5_ components fixed at their corresponding median level. (**F**) The BKMR model showed the estimated probability (95%CI) of lower creatinine-based eGFR in response to each-quantile increment in the mixture of PM_2.5_ components, compared to all PM_2.5_ components fixed at their corresponding median level.

As shown in Figure 5, WQS or QGC showed that the estimated β (95%CI) of eGFR in response to each 1-IQR increment in the mixture of PM_2.5_ components was −1.495 (−1. 816, −1.173) or −1.150 (−1.596, −0.705). BKMR showed that the estimated overall effect (95%CI) of eGFR in response to each 5-percentile increment in the mixture of PM_2.5_ components was −0.045 (−0.068, −0.022). The estimated ORs (95%CI) for lower creatinine-based eGFR in response to each 1-IQR increment in the PM_2.5_ component mixture were 1.183 (1.106, 1.266) for WQS and 1.182 (1.082, 1.291) for QGC. The BKMR models showed that the probability of lower creatinine-based eGFR increased with rising quantiles of the PM_2.5_ component mixture concentration when all PM_2.5_ components were fixed at their corresponding median levels, and the corresponding estimated effect was 0.003 (95%CI: 0.002, 0.004).

To identify the most influential components, we further examined the component-specific contributions. In the WQS models, SO_4_^2−^ dominated the weight for continuous eGFR (weight = 0.945, 95%CI: 0.764–0.999), whereas for categorical eGFR, the weights were more evenly distributed across multiple components with wide confidence intervals, indicating substantial uncertainty (Table S4). Consistently, the BKRM-derived PIPs identified SO_4_^2−^ as having the strongest association with both continuous and categorical eGFR, while BC showed the weakest evidence. The remaining components exhibited moderate PIPs ranging from 0.56 to 0.76, suggesting intermediate importance with some uncertainty (Table S5). Convergence diagnostics confirmed that the single MCMC chain used in the BKMR analysis mixed well, with stable fluctuations around a constant mean over 10,000 iterations and no apparent trends, indicating satisfactory convergence (Figure S4).

### 3.4. The Interaction Between PM_2.5_ Component Mixture and Nutritional Indices or Physical Activity in Relation to eGFR

As shown in Table 2, the joint associations of the PM_2.5_ component mixture with nutritional indices and leisure-time physical activity with lower creatinine-based eGFR were explored. The results show that, compared with individuals with high PNI and low PM_2.5_ component mixture exposure, the estimated ORs (95%CIs) for lower eGFR were 2.188 (1.674, 2.861) among those with low PNI and high PM_2.5_ component mixture exposure, 1.369 (1.028, 1.822) among those with high PNI and high PM_2.5_ component mixture exposure, and 1.995 (1.524, 2.613) among those with low PNI and low PM_2.5_ component mixture exposure. Similar joint effect patterns were observed for GNRI in combination with PM_2.5_ component mixture on lower creatinine-based eGFR. The results show that, compared with individuals with regular leisure-time physical activity and low PM_2.5_ component mixture exposure, the estimated ORs (95%CIs) for lower eGFR among those with irregular leisure-time physical activity and high PM_2.5_ component mixture exposure, irregular leisure-time physical activity and low PM_2.5_ component mixture exposure, and regular leisure-time physical activity and high PM_2.5_ component mixture exposure were 0.932 (0.723, 1.047), 0.810 (0.627, 1.047) and 0.932 (0.724, 1.201), respectively. No additive interaction was observed between the PM_2.5_ component mixture and either nutritional index or leisure-time physical activity, as indicated by the RERI and AP estimates (Table S5).

## 4. Discussions

This study indicates negative associations between exposure to PM_2.5_ and its components and lower creatinine-based eGFR, with OM showing a relatively larger weight in the PM_2.5_ component mixture-related lower creatinine-based eGFR. Exposure to a high level of the PM_2.5_ component mixture was related to lower creatinine-based eGFR. The different associations between PM_2.5_ component mixture and lower eGFR were found across subgroup by leisure-time physical activity and nutritional indices, but no significant interactive effect with nutritional indices or leisure-time physical activity on lower creatinine-based eGFR was observed.

A growing body of epidemiological evidence supports the negative associations between PM_2.5_ and its components and creatinine-based eGFR observed in this study. A comprehensive meta-analysis of over 3 million adults suggested that each 10 μg/m^3^ increment in short- and long-term PM_2.5_ exposure was associated with 0.57% and 0.90% reduction in eGFR values, respectively [43]. Beyond total mass, specific constituents have been implicated. The Veterans Affairs Normative Aging Study found that each-IQR increase in SO_4_^2−^ and lead was associated with eGFR reductions of 1.281 and 1.008 mL/min/1.73 m^2^, respectively [15]. Notably, the joint association of the PM_2.5_ component mixture is reinforced by the China Health and Retirement Longitudinal Study, which reported negative associations between the overall mixture and eGFR in 5696 middle-aged and older adults, with OM and SO_4_^2−^ showing particularly strong inverse associations [44]. This component-specific pattern extends to pediatric populations as well, as a national retrospective study in China found that NH_4_^+^ and NO_3_^−^ constituents had more pronounced associations with children’s eGFR than other chemical constituents [45]. Beyond general population studies, occupational exposure settings suggest that subway workers are exposed to PM_2.5_ enriched in metals (iron, manganese, and copper) derived from wheel and rail abrasion; elevated levels of these elements have been detected in their biological samples [46,47]. Given that both particulate mass and metal components have been individually linked to nephrotoxic effects, it is plausible that this metal-enriched exposure profile may have renal implications. Evidence has shown that subway-derived PM_2.5_ induces kidney inflammation, and occupational exposure to metal fumes has been associated with elevated urinary biomarkers of renal injury correlating with internal metal levels [47,48]. Since the inflammatory response occupational PM_2.5_ exposure is largely attributed to its metal content [47,48], these observations collectively suggest that subway workers may face an underrecognized renal risk beyond that captured by residential exposure to ambient PM_2.5_ and its components. Additionally, at the clinical outcome level, a large cohort study in the United States demonstrated that increased PM_2.5_ concentrations were associated with a 21–28% elevated risk of CKD onset and progression per 10 µg/m^3^ PM_2.5_ increment [49], while a multi-country meta-analysis reported a corresponding 15% higher CKD risk [50]. Biologically, the observed associations may be plausible through oxidative stress, inflammation, and endothelial dysfunction [51,52]. However, given the observational in nature, experimental studies further needed to elucidate the underlying biological pathways.

As the interaction did not reach statistical significance, the finding that the estimated association between PM_2.5_ component mixture and lower creatinine-based eGFR appeared more pronounced among individuals with lower nutritional indices rather than their counterparts should be interpreted with caution. Several pathways from prior literature may underlie this observation: malnutrition may reduce antioxidant defenses, impair renal repair, and enhance PM_2.5_-induced inflammation, possibly via deficiencies in antioxidant micronutrients [53,54,55]. However, as these mechanisms were not tested here, they remain speculative.

The interaction between PM_2.5_ component mixture and leisure-time physical activity in relation to lower creatinine-based eGFR did not achieve statistical significance. This finding is broadly consistent with previous reports. For instance, an analysis of 367,978 participants from the UK Biobank found no clear interactive effect between PM_2.5_ and physical activity on incident CKD [56]. Similarly, a longitudinal cohort study of 108,615 Taiwanese adults reported no significant interactive effect between chronic PM_2.5_ exposure and habitual physical activity on renal function decline (HR: 1.02, 95%CI: 0.97–1.07) or CKD development (HR: 1.00, 95%CI: 0.95–1.05) [57]. However, evidence also suggests that the benefits of physical activity may be attenuated under high levels of air pollution [23,24,25]. Regular physical activity may represent an accessible and low-cost strategy for health maintenance, particularly in relatively polluted areas [57,58]. The discrepancies across studies may be attributable to differences in the study population’s size, level of air pollution, and age distributions.

Several limitations should be noted. First, due to the cross-sectional study design, causal associations of exposure to PM_2.5_ and its components with lower creatinine-based eGFR could not be established. Furthermore, the compositional nature of PM_2.5_ exposure and the high collinearity among its components limit the causal interpretability of the component-specific estimates. Therefore, cautious interpretation of these exploratory findings is warranted. Second, given that creatinine-based eGFR may be influenced by muscle mass, diet, body size, and physical activity, the observed associations with nutritional indices should be interpreted with caution. In the future, studies incorporating cystatin *C*-based measurements are needed to confirm this study’s findings. Third, exposure misclassification may exist because individual air pollution exposure was estimated based solely on geocoded residential addresses. The exposure assessment did not capture workplace-specific exposure in subway environments such as workplace-specific PM_2.5_ components, time spent underground, job types, shift schedules, and ventilation conditions, likely underestimating true personal exposure levels. Several studies have shown that particulate matter levels in underground and subsurface subway stations are higher than those outdoors, indicating that using residential ambient PM_2.5_ exposure may underestimate individuals’ levels of PM_2.5_ exposure [59,60,61,62]. However, this misclassification is likely non-differential with respect to creatinine-based eGFR, which would attenuate the exposure–response associations toward the null. The occupational exposure burden should be further investigated using personal monitors and measured for metal-rich particles generated by rail and wheel abrasion, brake wear, welding or metal-cutting activities, tunnel dust, lubricants, and organic solvents. Fourth, although recent evidence suggests that GNRI and PNI may be useful nutritional indices in younger populations [32], these indices were originally developed for elderly individuals and patients with specific diseases, and prospective studies are still needed to validate their use in young working-age adults. Furthermore, as proxy markers derived from routine laboratory parameters, GNRI and PNI have not been formally validated in young working-age populations. Therefore, the findings should be considered exploratory and confirmed using validated nutritional assessment tools in future prospective study designs. Fifth, compared with excluded participants, the included sample was skewed toward older, male, less-educated, married, higher-income, and smoking/drinking individuals, limiting its representativeness. Moreover, as exclusions were non-random, the observed associations may be confounded by these baseline differences, potentially biasing the effect estimates in either direction (Table S6). Finally, although the observed reductions in creatinine-based eGFR were associated with the ambient residential PM_2.5_ component mixture, and should not be interpreted as clinically established kidney disease in this young population. Nonetheless, they warrant attention as preliminary findings that require validation through repeated measurements and additional markers of renal injury. Moreover, from a public health perspective, a small change at the individual level may correspond to a considerable burden at the population level, warranting attention to early prevention strategies.

## 5. Conclusions

Long-term exposure to residential ambient PM_2.5_ component mixture is associated with lower creatinine-based eGFR, and these associations appeared to differ across subgroups classified by nutritional indices or leisure-time physical activity. Given the descriptive nature of these subgroup findings, they should be viewed as exploratory and hypothesis-generating rather than conclusive. Future longitudinal studies with validated nutritional indices and comprehensive assessment of leisure-time physical activity are needed to corroborate these observations.

## Figures and Tables

**Table 1:** Baseline characteristics of the study population (*N* = 8477).

Variables	Total (*N* = 8477)	eGFR (mL/min/1.73 m^2^)		*p* Value
		≥90 (*n* = 7944)	<90 (*n* = 533)	
Age (years, mean ± SD)	27.18 ± 4.12	27.07 ± 4.04	28.77 ± 4.83	<0.001 ^a^
Gender (%)				<0.001 ^b^
Male	6808 (80.31)	6345 (79.87)	463 (86.87)	
Female	1669 (19.69)	1599 (20.13)	70 (13.13)	
Education level (years, *n*, %)				0.012 ^b^
<17	8331 (98.28)	7815 (98.38)	516 (96.81)	
≥17	146 (1.72)	129 (1.62)	17 (3.19)	
Marital status (*n*, %)				<0.001 ^b^
Married/living together	3060 (36.10)	2824 (35.55)	236 (44.28)	
Divorced/widowed/separated/Unmarried	5417 (63.90)	5120 (64.45)	297 (55.72)	
Income (RMB, *n*, %)				0.791 ^b^
<100,000	3728 (43.98)	3498 (44.03)	230 (43.15)	
100,000–	2471 (29.15)	2318 (29.18)	153 (28.71)	
≥150,000	2278 (26.87)	2128 (26.79)	150 (28.14)	
BMI (kg/m^2^, mean ± SD)	23.31 ± 3.66	23.26 ± 3.67	24.00 ± 3.47	<0.001 ^a^
Smoking status (*n*, %)				<0.001 ^b^
Never smoking	6331 (74.68)	5979 (75.26)	352 (66.04)	
Ever smoking	2146 (25.32)	1965 (24.74)	181 (33.96)	
Drinking status (*n*, %)				<0.001 ^b^
Never	6762 (79.77)	6373 (80.22)	389 (72.98)	
Current	1621 (19.12)	1485 (18.69)	136 (25.52)	
Former	94 (1.11)	86 (1.08)	8 (1.50)	
Leisure-time physical activity (*n*, %)				<0.001 ^b^
Yes	4774 (56.32)	4515 (56.84)	259 (48.59)	
no	3703 (43.68)	3429 (43.16)	274 (51.41)	
CVD factors (*n*, %)				<0.001 ^b^
No	5503 (64.92)	5204 (65.51)	299 (56.10)	
Yes	2974 (35.08)	2740 (34.49)	234 (43.90)	
Nutritional indices				
GNRI (median, IQR)	108.94 (105.73, 111.83)	109.00 (105.75, 111.92)	108.22 (105.13, 110.79)	<0.001 ^c^
PNI (median, IQR)	56.70 (54.20, 59.20)	56.75 (54.25, 59.30)	55.70 (53.30, 57.95)	<0.001 ^c^
Particulate matter (median, IQR)				
PM_2.5_	53.61 (51.27, 61.04)	53.29 (51.27, 61.03)	55.09 (52.47, 61.76)	<0.001 ^c^
SO_4_^2−^	9.63 (8.69, 10.04)	9.51 (8.69, 10.04)	9.72 (8.76, 10.09)	<0.001 ^c^
NO_3_^−^	11.58 (10.76, 12.52)	11.58 (10.76, 12.52)	11.65 (10.78, 12.61)	0.021 ^c^
NH_4_^+^	6.93 (6.46, 7.56)	6.93 (6.46, 7.56)	7.01 (6.49, 7.64)	0.016 ^c^
OM	13.51 (13.17, 15.47)	13.51 (13.17, 15.47)	13.77 (13.23, 16.39)	0.001 ^c^
BC	2.62 (2.51, 3.01)	2.62 (2.49, 2.99)	2.64 (2.57, 3.16)	0.001 ^c^

IQR: interquartile range; BMI: body mass index; GNRI: Geriatric Nutritional Risk Index; PNI: Prognostic Nutritional Index; PM_2.5_: fine particulate matter; sulfate: SO_4_^2−^; nitrate: NO_3_^−^; ammonium: NH_4_^+^; BC: black carbon; organic matter: OM. ^a^ Student’s *t* test was used to compare normally distributed continuous variables between individuals with eGFR < 90 and ≥90 mL/min/1.73 m^2^; ^b^ A Chi-square test was used to test the distributions of categorical variables between individuals with eGFR < 90 and ≥90 mL/min/1.73 m^2^; ^c^ Mann–Whitney U test was used to test non-normally distributed continuous variables between individuals with eGFR < 90 and ≥90 mL/min/1.73 m^2^.

**Table 2:** The joint associations between PM_2.5_ component mixture and either nutritional indices or leisure-time physical activity with lower eGFR ^a^.

Variables	Cases/Total Participants	OR (95%CI)
Low PM_2.5_ component mixture + High PNI ^a^	91 (2187)	Reference
High PM_2.5_ component mixture + High PNI ^a^	111 (2003)	1.369 (1.028, 1.822)
Low PM_2.5_ component mixture + Low PNI ^a^	165 (2140)	1.995 (1.524, 2.613)
High PM_2.5_ component mixture + Low PNI ^a^	166 (2147)	2.188 (1.674, 2.861)
Low PM_2.5_ component mixture + High GNRI ^a^	103 (2234)	Reference
High PM_2.5_ component mixture + High GNRI ^a^	124 (2004)	1.395 (1.064, 1.828)
Low PM_2.5_ component mixture + Low GNRI ^a^	153 (2093)	1.803 (1.379, 2.356)
High PM_2.5_ component mixture + Low GNRI ^a^	153 (2146)	1.889 (1.451, 2.460)
Low PM_2.5_ component mixture + Regular leisure-time physical activity ^b^	131 (1921)	Reference
High PM_2.5_ component mixture + Regular leisure-time physical activity ^b^	143 (1782)	0.932 (0.724, 1.201)
Low PM_2.5_ component mixture + Irregular leisure-time physical activity ^b^	125 (2406)	0.810 (0.627, 1.047)
High PM_2.5_ component mixture + Irregular leisure-time physical activity ^b^	134 (2368)	0.932 (0.723, 1.047)

GNRI: Geriatric Nutritional Risk Index; PNI: Prognostic Nutritional Index. ^a^ Adjusted for age, gender, socioeconomic status (education levels, annual family income, marital status), lifestyle (smoking and drinking status), leisure-time physical activity, BMI and CVD risk factors. ^b^ Adjusted for age, gender, socioeconomic status (education levels, annual family income, marital status), lifestyle (smoking and drinking status), BMI and CVD risk factors. PM_2.5_ component mixture and nutritional indices were classified into low and high based on their corresponding median level.

## Data Availability

The raw data supporting the conclusions of this article will be made available by the authors on request.

## Supplementary Materials

The following supporting information can be downloaded at https://www.mdpi.com/article/10.3390/nu18152580/s1, Figure S1: Flow chart of participant selection and exclusion; Figure S2: Associations of PM_2.5_ and its components with kidney function; Figure S3: Associations of PM_2.5_, its components, and nutritional indices with eGFR after excluding individuals with eGFR < 90 mL/min/1.73 m^2^; Figure S4: Trace plot of the model parameter for association between PM_2.5_ components and continuous (A) and categorical eGFR (B), across 10,000 MCMC iterations; Table S1: Associations of PM_2.5_ and its components, nutritional indices with blood urea nitrogen and serum creatinine; Table S2: Quantile regression analysis of NO_3_^−^ and NH_4_^+^ exposure with eGFR ^a^; Table S3: Additive interaction of PM_2.5_ component mixture, nutritional indices and physical activity on lower kidney function ^a^; Table S4: The weights and 95%CI of the PM_2.5_ components in the WQS model ^a^; Table S5: The PIPs of the PM2.5 components in the BKMR model; Table S6: Comparison of baseline characteristics between included and excluded participants.

## Abbreviations

95%CI: confidence interval; BKMR: Bayesian Kernel Machine Regression; BMI: body mass index; BC: black carbon; CKD: chronic kidney disease; eGFR: estimated glomerular filtration rate; GNRI: Geriatric Nutritional Risk Index; NH_4_^+^: ammonium; NO_3_^−^: nitrate; IQR: interquartile range; OM: organic matter; PM_2.5_: particulate matter with aerodynamic diameter ≤ 2.5 µm; PNI: Prognostic Nutritional Index; QGC: Quantile-based G-Computation; SO_4_^2−^: sulfate; TAP: Tracking Air Pollution; SD: standard deviation; WQS: weighted quantile sum.
