# Supplementary material for: Joint Associations of Residential Ambient PM2.5 Components, Nutritional Indices, and Leisure-Time Physical Activity with Lower Estimated Glomerular Filtration Rate Among Subway Workers
Source: Nutrients. 2026 Aug 6;18(15):2580. doi: 10.3390/nu18152580 (PMC13468558; doi:10.3390/nu18152580)
Supplement: Supplementary file 1 [file nutrients-18-02580-s001.zip › nutrients-4411394-supplementary.pdf]

Figure S1 Flow chart of participant selection and exclusion.

Figure S2 Associations of $PM_{2.5}$ and its components with kidney function. Variables highlighted in green with a "►" symbol represent the exposure; variables in blue with an "I" denote the outcomes. Blue variables also serve as antecedents of the outcome, while red variables are antecedents of both the outcome and the exposure.

Figure S3 Associations of $PM_{2.5}$, its components, and nutritional indices with eGFR after excluding individuals with eGFR < 90 mL/min/1.73 m². The estimated β coefficients and their corresponding 95% confidence intervals (CIs) were evaluated using the following models: Model 1 was unadjusted. Model 2 was adjusted for age and sex. Model 3 was adjusted for age, gender, socioeconomic status (education levels, annual family income, marital status), lifestyles (smoking and drinking status), leisure-time physical activity, BMI and CVD risk factors.

Figure S4 Trace plot of the model parameter for association between $PM_{2.5}$ components and continuous (A) and categorical eGFR (B), across 10,000 MCMC iterations. The x-axis represents the iteration number, and the y-axis shows the sampled parameter values.

Supplementary Table S1 Associations of $PM_{2.5}$ and its components, nutritional indices with blood urea nitrogen and serum creatinine.

Variables	Blood urea nitrogen (β, 95%CI)	Serum creatinine (β, 95%CI)
$PM_{2.5}$	-0.056 (-0.152, 0.04)	1.255 (0.904, 1.605)
SO_4^{2-}	-0.069 (-0.143, 0.005)	1.228 (0.959, 1.498)
NO_3^-	-0.036 (-0.123, 0.05)	0.986 (0.671, 1.302)
NH_4^+	-0.036 (-0.12, 0.048)	0.918 (0.610, 1.226)
OM	-0.032 (-0.112, 0.047)	0.830 (0.539, 1.121)
BC	-0.037 (-0.123, 0.049)	0.925 (0.611, 1.239)
GNRI	0.088 (0.074, 0.103)	-0.410 (-0.463, -0.357)
PNI	0.094 (0.078, 0.111)	-0.404 (-0.464, -0.344)

Abbreviations: GNRI: Geriatric Nutritional Risk Index; PNI: Prognostic Nutritional Index; Model 1 was Unadjusted. Model 2 was adjusted for age and gender. Model 3 was adjusted for age, gender, socioeconomic status (education levels, annual family income, marital status), lifestyles (smoking and drinking status), leisure-time physical activity, BMI and CVD risk factors.

Table S2 Quantile regression analysis of NO_3^- and NH_4^+ exposure with eGFR[a]

Variable	Quantile	Beta (95%CI)	*P* value	Variable	Quantile	Beta (95%CI)	*P* value
NO_3^-	10%	-0.63 (-0.99, -0.26)	<0.001	NH_4^+	10%	-0.92 (-1.48, -0.37)	0.001
NO_3^-	20%	-0.58 (-0.95, -0.22)	0.002	NH_4^+	20%	-0.88 (-1.46, -0.31)	0.003
NO_3^-	30%	-0.86 (-1.21, -0.51)	<0.001	NH_4^+	30%	-1.25 (-1.76, -0.74)	<0.001
NO_3^-	40%	-1.02 (-1.42, -0.62)	<0.001	NH_4^+	40%	-1.54 (-2.17, -0.91)	<0.001
NO_3^-	50%	-1.19 (-1.63, -0.76)	<0.001	NH_4^+	50%	-1.74 (-2.43, -1.06)	<0.001
NO_3^-	60%	-1.19 (-1.61, -0.77)	<0.001	NH_4^+	60%	-1.87 (-2.52, -1.22)	<0.001
NO_3^-	70%	-1.52 (-2.08, -0.97)	<0.001	NH_4^+	70%	-2.25 (-3.18, -1.33)	<0.001
NO_3^-	80%	-1.18 (-1.96, -0.41)	0.003	NH_4^+	80%	-1.81 (-3.06, -0.57)	0.004
NO_3^-	90%	-1.51 (-2.21, -0.80)	<0.001	NH_4^+	90%	-2.17 (-3.34, -1.00)	<0.001

Abbreviations: Beta: regression coefficients; *OR*: Odds Ratio; CI: Confidence Interval; nitrate: NO_3^-; ammonium: NH_4^+; [a] Adjusted for age, gender, socioeconomic status (education levels, annual family income, marital status), lifestyles (smoking and drinking status), leisure-time physical activity, BMI and CVD risk factors.

Table S3 Additive interaction of $PM_{2.5}$ component mixture, nutritional indices and physical activity on lower kidney function [a].

Variables	Effect Modifier	RERI	AP
Air pollution mixtures	GNRI [a]	-0.176 (-0.775, 0.423)	-0.080 (-0.355, 0.195)
	PNI [a]	-0.308 (-0.877, 0.261)	-0.163 (-0.467, 0.141)
	Leisure-time physical activity[b]	0.174 (-0.258, 0.606)	0.111 (-0.161, 0.382)

Abbreviations: GNRI: Geriatric Nutritional Risk Index; PNI: Prognostic Nutritional Index; [a] Adjusted for age, gender, socioeconomic status (education levels, annual family income, marital status), lifestyles (smoking and drinking status), leisure-time physical activity, BMI and CVD risk factors; [b]Adjusted for age, gender, socioeconomic status (education levels, annual family income, marital status), lifestyles (smoking and drinking status), BMI and CVD risk factors.

Table S4 The weights and 95% CI of the $PM_{2.5}$ components in the WQS model[a]

Variables	Continuous eGFR		Categorical eGFR
SO_4^{2-}	0.945 (0.764, 0.999)	OM	0.312 (0, 1)
BC	0.024 (0.0001, 0.101)	NO_3^-	0.244 (0, 0.935)
OM	0.012 (0.0001, 0.111)	NH_4^+	0.238 (0, 1)
NH_4^+	0.015 (0.0001, 0.073)	BC	0.201 (0, 1)
NO_3^-	0.004 (0.0001, 0.039)	SO_4^{2-}	0.004 (0, 0.034)

eGFR: estimated glomerular filtration rate; $PM_{2.5}$: fine particulate matter; sulfate: SO_4^{2-}; nitrate: NO_3^-; ammonium: NH_4^+; BC: black carbon; organic matter: OM; WQS: weighted quantile sum. [a]adjusted for age, gender, socioeconomic status (education levels, annual family income, marital status), lifestyles (smoking and drinking status), leisure-time physical activity, BMI and CVD risk factors.

Table S5 The PIPs of the $PM_{2.5}$ components in the BKMR model

variable	Continuous eGFR	variable	Categorical eGFR
SO_4^{2-}	0.9966	SO_4^{2-}	1
NH_4^+	0.7552	OM	0.9988
NO_3^-	0.5896	NO_3^-	0.9728
OM	0.5556	NH_4^+	0.851
BC	0.4744	BC	0.8148

BKMR: Bayesian Kernel Machine Regression; eGFR: estimated glomerular filtration rate; $PM_{2.5}$: fine particulate matter; PIPs: posterior inclusion probabilities; sulfate: SO_4^{2-}; nitrate: NO_3^-; ammonium: NH_4^+; BC: black carbon; organic matter: OM. [a]adjusted for age, gender, socioeconomic status (education levels, annual family income, marital status), lifestyles (smoking and drinking status), leisure-time physical activity, BMI and CVD risk factors.

Table S6 Comparison of baseline characteristics between included and excluded participants.

Variables	Total(N=11960)	Exclude(N=3483)	Include(N=8477)	*P* value
Age (years, Mean ± SD)	26.64 ±4.36	25.41±4.68	27.18 ±4.12	<0.001 [a]
Gender (n, %)				<0.001 [b]
Male	9338 (78.08)	2531 (72.67)	6808 (80.31)	
Female	2622 (21.92)	952 (27.33)	1669 (19.69)	
Education level (years, n, %)				<0.001 [b]
< 17	11435 (95.61)	3104 (89.12)	8331 (98.28)	
≥17	367 (3.07)	221 (6.34)	146 (1.72)	
Marital status (n, %)				<0.001 [b]
Married/living together	3788 (31.67)	728 (20.90)	3060 (36.10)	
Divorced/widowed/separated/Unmarried	8088 (67.63)	2671 (76.68)	5417 (63.90)	
Income (RMB, n, %)				<0.001 [b]
<100000	5289 (44.22)	1562 (44.85)	3728 (43.98)	
100000-	3170 (26.51)	700 (20.10)	2471 (29.15)	
≥ 150000	3049 (25.49)	769 (22.08)	2278 (26.87)	
Smoking status (n, %)				<0.001 [b]
Never smoking	9134 (76.6)	2803 (81.48)	6331 (74.68)	
Ever smoking	2791(23.4)	645 (18.52)	2146 (25.32)	
Drinking status (n, %)				0.046 [b]
Never	9584 (80.13)	2822 (81.02)	6762 (79.77)	
Current	2112 (17.66)	491 (14.10)	1621 (19.12)	
Former	233 (1.95)	139 (3.99)	94 (1.11)	

[a] Student's t test was used to compare normally distributed continuous variables between excluded and included individuals groups; [b]A Chi-square test was used to test the distributions of categorical variables between excluded and included individuals groups.

Figure S1

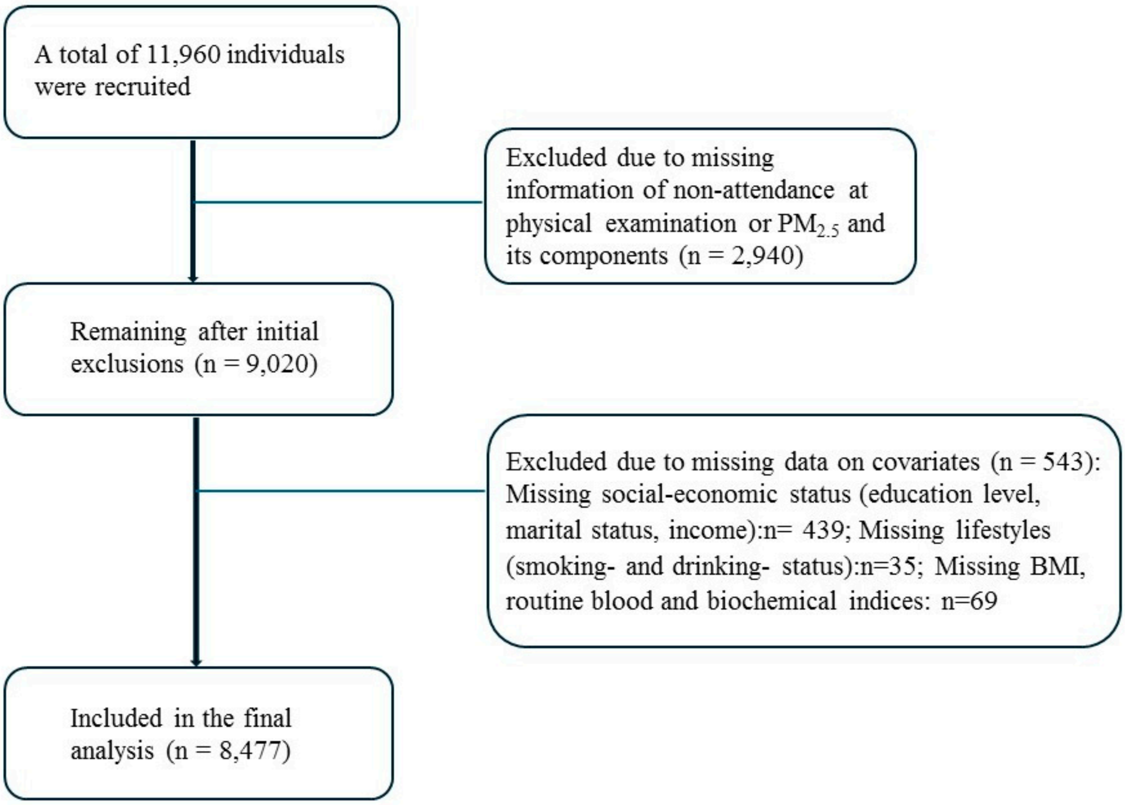
A total of 11,960 individuals were recruited
Excluded due to missing information of non-attendance at physical examination or $PM_{2.5}$ and its components (n = 2,940)
Remaining after initial exclusions (n = 9,020)
Excluded due to missing data on covariates (n = 543): Missing social-economic status (education level, marital status, income):n= 439; Missing lifestyles (smoking- and drinking- status):n=35; Missing BMI, routine blood and biochemical indices: n=69
Included in the final analysis (n = 8,477)

Figure S2

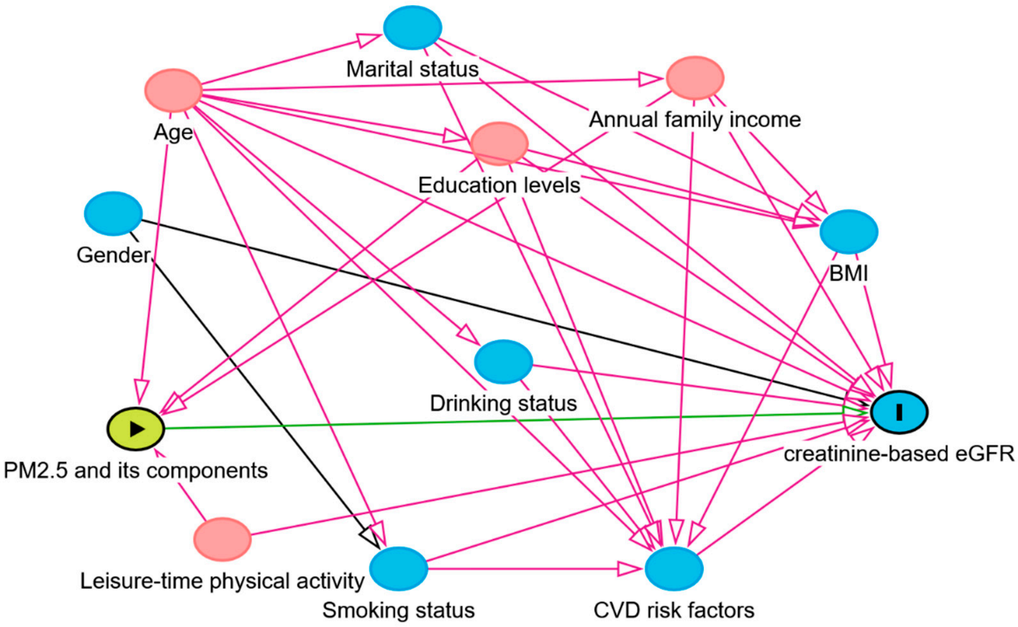
Marital status
Annual family income
Age
Education levels
Gender
BMI
Drinking status
PM2.5 and its components
creatinine-based eGFR
Leisure-time physical activity
Smoking status
CVD risk factors

Figure S3

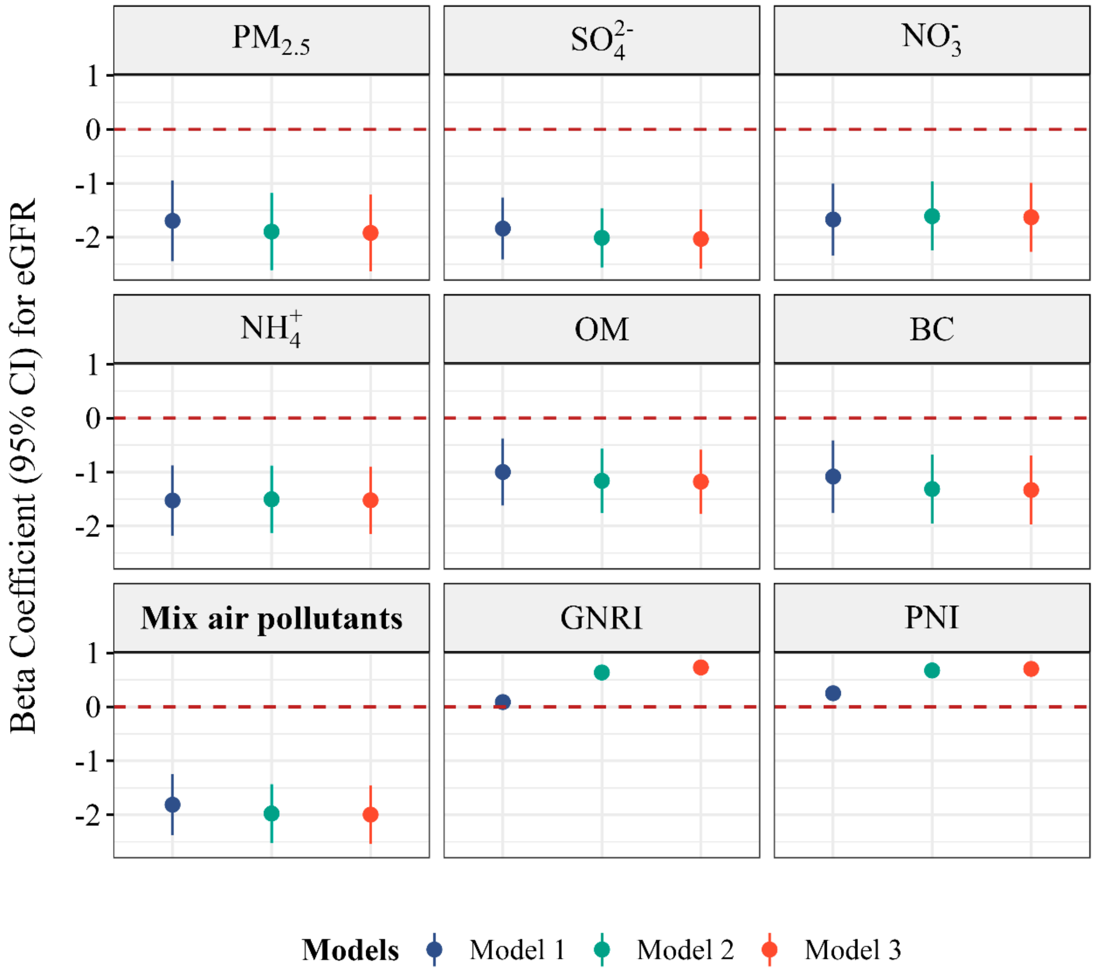

$PM_{2.5}$
SO_4^{2-}
NO_3^-
NH_4^+
OM
BC
Mix air pollutants
GNRI
PNI
1
0
-1
-2
Beta Coefficient (95% CI) for eGFR
Models
Model 1
Model 2
Model 3

Figure S4

A

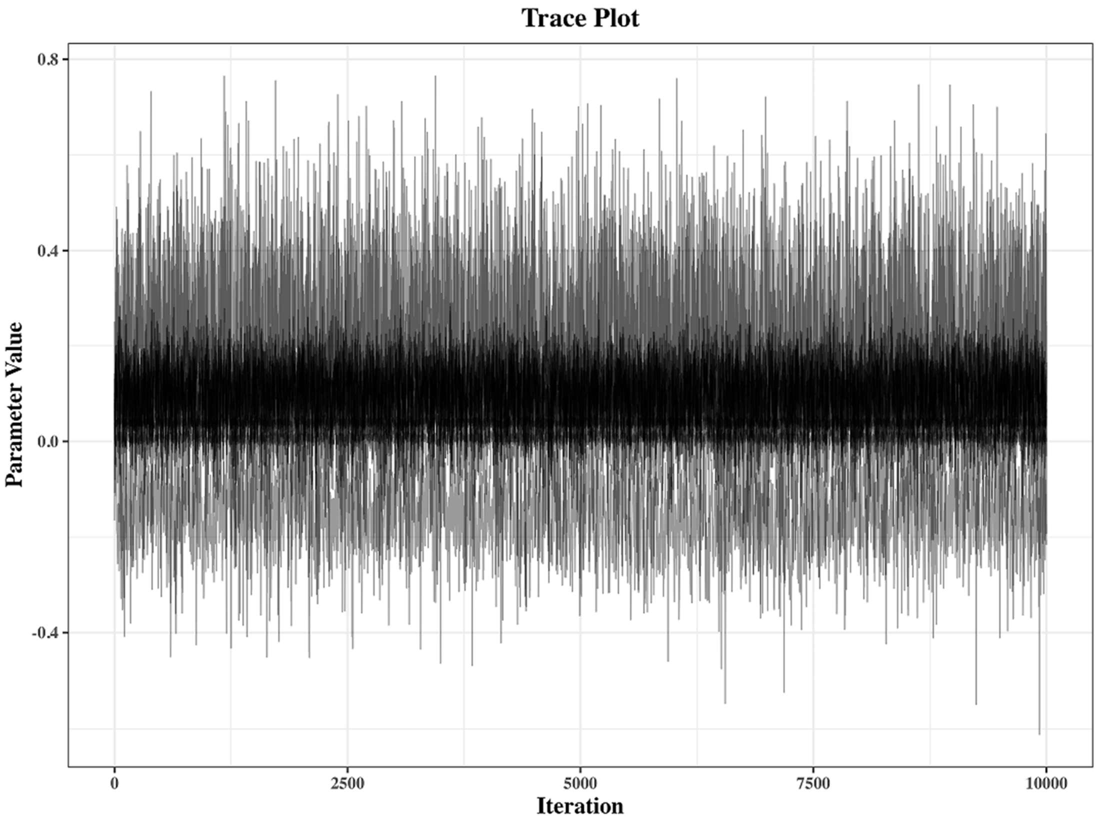
Trace Plot
0.8
0.4
0.0
-0.4
Parameter Value
0
2500
5000
7500
10000
Iteration

B

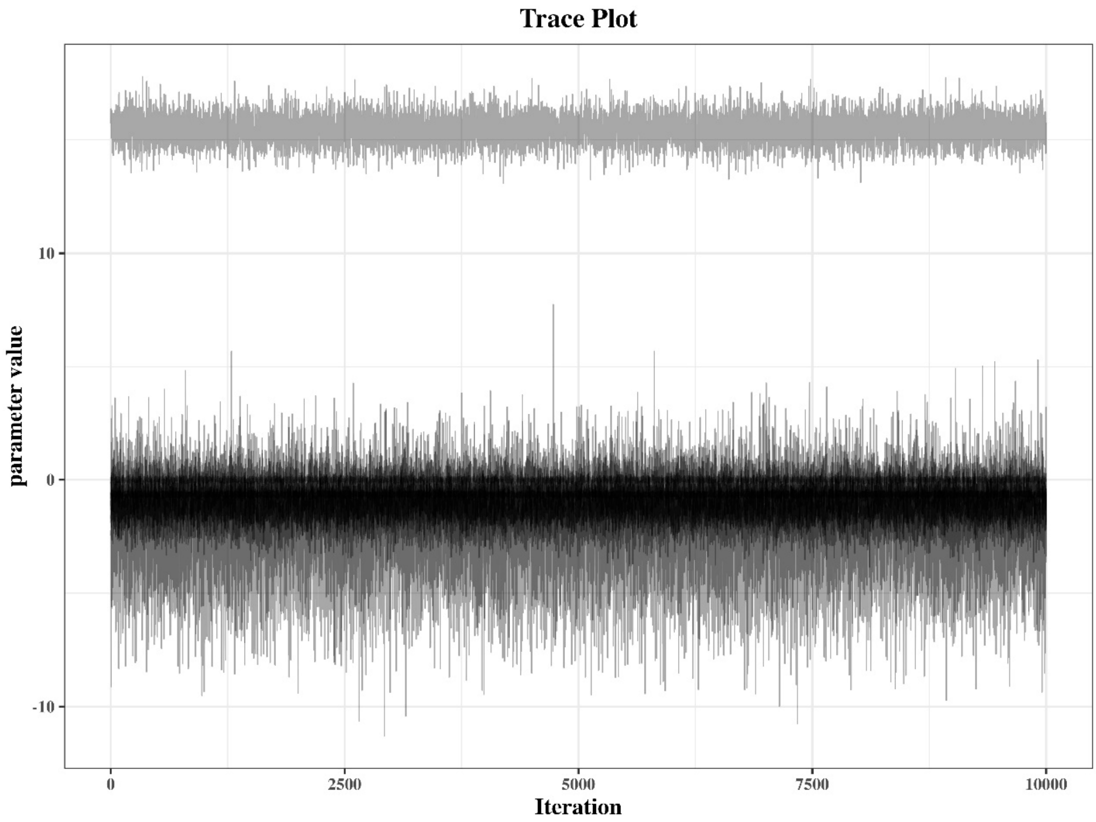
Trace Plot
10
0
-10
parameter value
0
2500
5000
7500
10000
Iteration
